# Effect of Alkali Salts on the Hydration Process of Belite Clinker

**DOI:** 10.3390/ma15103424

**Published:** 2022-05-10

**Authors:** Valeriia Iliushchenko, Lukáš Kalina, Martin Sedlačík, Vladislav Cába, Jiří Másilko, Radoslav Novotný

**Affiliations:** Faculty of Chemistry, Institute of Materials Science, Brno University of Technology, 612 00 Brno, Czech Republic; kalina@fch.vut.cz (L.K.); xcsedlacik@vutbr.cz (M.S.); vladislav.caba@vut.cz (V.C.); masilko@fch.vut.cz (J.M.); xcnovotny2@fch.vut.cz (R.N.)

**Keywords:** belite-rich cement, alkaline admixtures, hydration, phase composition

## Abstract

Belite-rich cement is a low carbon footprint binder. However, its use is accompanied by a low initial rate of hydration. This can be partially eliminated by grinding to a high specific surface or through the addition of admixtures (mineralizators or activators). The influence of alkaline activators CaSO_4_, Na_2_SO_4_ and Na_2_CO_3_ (in the amount of 5 wt.% related to the clinker weight) on the hydration course, as well as the quantity of hydration products in belite-rich cements, were investigated in this paper. Belite-rich clinker was laboratory-synthetized and ground together with activators to prepare various belite-rich cements. Next, the hydration kinetics and the hydrated phase assemblage were investigated using isothermal calorimetry, X-ray powder diffraction and thermogravimetric and differential thermal analyses. The use of selected admixtures allowed us to obtain belite-rich cements with higher hydraulic activity in the initial period.

## 1. Introduction

Ordinary Portland cement (OPC) is one of the most used materials on a global scale, the production of which has continually increased due to rising population, urbanization and infrastructure development [1]. Unfortunately, its manufacturing process is accompanied by a significant demand for energy and by high CO_2_ emissions. Nowadays, the cement industry is the third largest industrial energy consumer and is responsible for 6–7% of global CO_2_ emissions [2]. To reduce these negative aspects, supplementary cementitious materials (SCM) are widely used to replace Portland clinker. Another important direction also lies in the production of types of cement with a lower energy requirement in comparison to OPC. Belite cement represents such an alternative.

Belite clinker usually contains more than 40% belite and a low amount of alite (less than 35%), as opposed to OPC [3]. Elevated calcium disilicate (C_2_S) content results in a lime saturation factor (LSF) of less than 80 [4]. Therefore, its production is based on the burning process of raw meal with reduced CaCO_3_ content, and, simultaneously, the burning temperature can be about 100 °C lower than the average for OPC. On the other hand, the greater hardness of belite compared to alite requires more electrical energy for grinding to the same fineness as OPC.

In terms of application properties, belite-rich cements provide lower water demand, better compatibility with most plasticizing admixtures, lower heat evolution and lower drying shrinkage compared to OPC [3]. Last but not least, belite cements have also been shown to have better durability in terms of resistance to sulfates [5] and acids [6], mainly due to a smaller amount of portlandite in the hydration products. Unfortunately, these advantages are overshadowed by lower early strength development.

To obtain acceptable strength in the initial stages of hydration, the LSF value should reach levels at which some alite can still be formed. Alternatively, belite cements containing alite-free clinker could be mixed with ordinary Portland clinker as a source of the required quantity of alite [7]. The strength characteristics are also affected by additives in the raw meal, where the substitution of Ba^2+^ for Ca^2+^ ions [8], as well as suitable doping with combinations of sulfur and lithium [9], can significantly increase C_2_S reactivity. An effective enhancement of belite reactivity may be achieved by increasing alkali content either in the raw meal or, subsequently, in the cement itself. The former case is based on the alkaline stabilization of the most reactive polymorphs of dicalcium silicate, α’-C_2_S, and α-C_2_S at room temperature. It was confirmed that such activated cement shows a higher degree of hydration in the initial stages [10,11]. The latter case is caused by the usage of suitable alkaline admixtures. It has been found that the presence of Na-activators in the form of NaOH, Na_2_SO_4_, or Na_2_CO_3_ accelerates β-C_2_S hydration [12,13]. Similar findings have also been reported for activation by sodium or potassium hydroxide [14]. Moreover, it has been proved that alkali content influences the hydraulicity of γ-C_2_S, which is otherwise almost inactive in water [15].

Based on these findings, alkali ions play a very important role in the hydration process of belite-rich cement. Therefore, this paper is focused on the characterization of its hydration process in the presence of alkali salts. A series of analyses, including XRD, TG-DTA and calorimetry measurements, were applied to explain the reaction mechanisms of alkaline admixtures (i.e., Na_2_SO_4_, and Na_2_CO_3_), which were added to the system instead of gypsum as a setting regulator. Compared to other, related studies, this investigation describes the role of alkali salts in terms of belite clinker hydration, not only their influence on pure phases. From this point of view, the obtained results can provide useful insights for the practical applications of belite cement activation possibilities. 

## 2. Materials and Methods

Raw mix for the belite clinker (BC) burning process was prepared by mutual milling commercial raw mix used for Portland clinker production (Mokrá cement plant, HeidelbergCement Group, Brno-jih, Czech Republic) with the specific addition of diatomaceous earth (LB Minerals, s.r.o., Horní Bříza, Czech Republic) serving as a dopant of SiO_2_. Based on the preliminary experiments, a C/S ratio of 3.55 was chosen. The phase composition of the prepared clinker was as follows: β-C_2_S (72%), γ-C_2_S (9%), C_3_A (calcium aluminate) (4%), C_3_S (tricalcium silicate) (9%) and brownmillerite (5%). Its chemical composition is summarized in Table 1. The raw mix was compressed into tablets at a pressure of 10 MPa. The tablets were fired at 1300 °C with a ramp of 5 °C/min and a holding time of 45 min in an elevator furnace. Then, the furnace was opened, and the tablets were removed and cooled in water with ice. After cooling, the tablets were dried in an oven at 105 °C. The prepared clinker was mixed with the alkaline admixture or gypsum and ground in a vibrating mill to a Blaine fineness of 350 m^2^/kg. The amount of alkali salts was calculated as the molar equivalent to the common addition of gypsum in OPC (5% by weight of clinker). Anhydrous sodium sulfate and sodium carbonate (Penta, s.r.o., Prague, Czech Republic) were used as alkaline admixtures in this study.

Cement pastes based on belite clinker were prepared to study the hydration processes. The water/cement ratio (w/c) was set to 0.50 for all pastes. A summary of the pastes’ compositions is provided in Table 2. The fresh pastes were used for subsequent testing.

The evolution of hydration heat was monitored by means of TAM Air isothermal calorimeter (TA instruments, New Castle, DE, USA). The samples containing 5.00 ± 0.01 g of belite cement were weighed and uniformly distributed into the PE vials (20 mL). The reference material was demineralized water in an amount corresponding to the heat capacity of the sample. All cement components, vials, and references were tempered in a calorimeter for several hours at a constant temperature of 25 °C ± 0.02 °C. Subsequently, the mixing water was added to the cement with aforementioned w/c of 0.50 and stirred for 2 min. The heat evolution was recorded as the heat flow (mW/g) immediately after. 

X-ray powder diffraction (PANanalytical Empyrean, Almelo, The Netherlands) of belite clinker was measured using CuKα radiation equipped with a 3D detection system, PIXcel3D. Samples were step scanned from 5° to 80° 2θ using vertical high-resolution goniometer with the step size 0.013° 2θ. The crystallographic evaluation and quantitative analysis were performed by the Rietveld method in Highscore software. The crystalline phases identified in BC were C_3_S, β-C_2_S, C_4_AF and C_3_A. The same method of measurement was used for phase composition determination of hydrated belite-rich cement samples in various steps of hydration, selected according to calorimetric curves.

The hydration process was stopped by immersing samples in 100 mL of isopropanol for 15 min. Samples were then rinsed once with isopropanol, twice with diethyl ether, and dried at 40 °C. The hydration products were studied using the aforementioned XRD and TG-DTA analysis (SDT650, TA instruments, New Castle, DE, USA). The measurement was carried out with 40–50 mg samples in dried air atmosphere with a heating rate of 20 °C/min up to 1000 °C.

Scanning electron microscopy was used to characterize and examine the fracture of the surface of hydrated pieces (28 days) in backscattered electron mode using ZEISS EVO LS10 (Carl Zeiss NTS, Jena, Germany) electron microscope with an energy dispersive analyzer OXFORD X-Max 80 mm^2^ (Oxford Instruments, Plc., Abingdon, UK). Samples were stuck onto carbon tape, and the exposed fractured surfaces were sputter-coated with gold. The working distance in the measurement process was set to 12 mm, and the accelerate voltage was 15 kV.

## 3. Results and Discussion

### 3.1. Hydration Process

**Figure 1:**
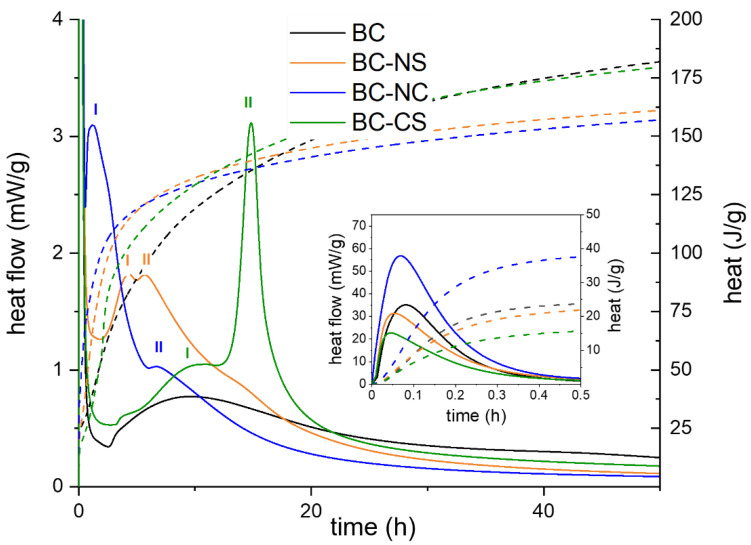
The dependence of heat flow and total heat on time during the first 48 h of hydration for various belite-rich cements. Dot line is the total heat realized. Peaks I and II mean silicate and the peak belong to the relevant alkaline activator.

Undoubtedly, the most crucial method of monitoring changes in hydration processes that are ongoing in samples based on belite clinker with various admixtures is isothermal calorimetry. This analytical technique was applied to investigate the hydration stages of belite cements by measuring the heat release coming, predominantly, from chemical reactions. The results of the isothermal calorimetric measurement of the belite-rich cement samples containing alkaline admixtures during the first 48 h of hydration are shown in Figure 1. It is obvious that all calorimetric curves are characterized by several main periods having different courses for particular samples. A closer look into the reaction mechanism within these periods is further described in more detail in the following sections. 

#### 3.1.1. Initial Period

In the initial period in the first minutes after mixing with water, the most important processes are the wetting of cement grains and a rapid movement of ions from their surface into the solution [16]. A significant amount of heat is released, especially due to the congruent dissolution of C_3_S, while the β-C_2_S hydration process is considerably slower [16,17]. However, the heat flows for individual samples differ significantly in this region. The presence of sulfates plays an important role in this case. In the absence of gypsum (sample BC), C_3_A reacts quickly with water, resulting in the formation of hexagonal calcium aluminates hydrates such as C_4_AH_19_, C_4_AH_13_, and C_2_AH_8_, associated with a high release of heat [18,19,20]. On the other hand, the addition of gypsum (sample BC-CS) promotes the precipitation of ettringite, but the reaction with aluminate phases is quite short and directly followed by a deceleration [21]. This substantial slowdown was previously attributed to surface coating by ettringite [22,23]. However, the current research explains the retardation mechanism via the adsorption of sulfate ions on the active sites of C_3_A [17,24]. Regardless of the aforementioned suggested mechanisms, the total heat evolution during the initial hydration period of the gypsum-containing sample was significantly lower. Based on these findings, a similar course for the initial period can be expected when gypsum is replaced by Na_2_SO_4_ (NS) with a same molar concentration of SO_4_^2−^ ions. However, the total heat flow in this period was almost the same as in the system without any admixtures. The obtained results suggest that the sole presence of sulfate ions is not a dominant factor in controlling the kinetics of aluminate phase hydration. This phenomenon was also confirmed by previous studies [23,25], explaining that only the coexistence of calcium and sulfate ions plays a critical role in the retardation of C_3_A hydration. Nevertheless, the highest amount of heat evolution was observed in Na_2_CO_3_ (NC) activation in the first 30 min. The main reason for such an increase in heat flow is predominantly related to the dissolution of Na_2_CO_3_. The dissolution process is highly exothermic in the case of Na_2_CO_3_ (ΔH_diss_ = −26.7 kJmol^−1^) in comparison to CaSO_4_·2H_2_O (CS) (ΔH_diss_ = 0.6 kJmol^−1^) or Na_2_SO_4_ (ΔH_diss_ = 1.5 kJmol^−1^) at 25 °C, with respect to the molality of the resulting solutions [26,27].

#### 3.1.2. Induction Period

After the initial period, a drop-in heat activity was observed, which corresponded with the induction period. This region represents the time to reach the critical size of C–S–H nuclei and to start their growth [28]. It suggests that the range of the induction period certainly corresponds mainly with calcium silicate phase dissolution due to ionic concentrations in the aqueous phase [21]. The dissolution rate can be fundamentally affected by the addition of specific admixtures. In general, alkali salts belong to the group of chemical compounds acting as accelerators and tend to promote the dissolution process of silicates [29]. The mechanism of this effect lies in the precipitation of insoluble calcium salts, which generally increases the dissolution of calcium silicates by keeping the calcium concentration at a low level [30]. Consequently, the pH of the pore solution is increased by the formation of alkali hydroxide, and a higher concentration of dissolved silica species is detected, which should precipitate as C–S–H gel as soon as the calcium concentration starts to rise again. Moreover, the alkali hydroxide environment lowers the calcium concentration required to reach the maximum calcium hydroxide supersaturation at which the onset of the acceleration period begins [16]. A noticeable shortening of the induction period resulting in a faster precipitation of C–S–H gel was observed after the addition of Na_2_CO_3_ as well as Na_2_SO_4_.

#### 3.1.3. Acceleration Period

As mentioned above, the main precipitation of C–S–H gel characterized by heat release corresponds to the so-called acceleration period. The evolution of heat in this region suggests that the highest amount of C–S–H products is formed by acceleration with Na_2_CO_3_. Activation with Na_2_SO_4_ also shows a noticeable acceleration of the hydration of silicate phases (predominantly, C_3_S) compared both to systems with added gypsum and belite clinker itself.

#### 3.1.4. Deceleration Period

The typical silicate peak (I) in OPC systems is shortly followed by another peak (II) corresponding to the sulfate depletion point falling within the deceleration period. In this time period, the concentration of sulfates in solution is exhausted and the renewed dissolution of C_3_A and the formation of ettringite takes place [31]. This reaction mechanism can also be attributed to belite clinker with added gypsum.

#### 3.1.5. Final Slow Reaction Period

During this period, activity is low, because the rate of the hydration becomes diffusion-controlled. The C–S–H gel is still formed due to the continuing hydration of silicate phases and may be densified. Whereas the hydration kinetics of β-C_2_S at laboratory temperature is low compared to other clinker minerals, the contribution of β-dicalcium silicate hydration can be expected. 

### 3.2. X-Ray Diffraction Analysis

**Figure 2:**
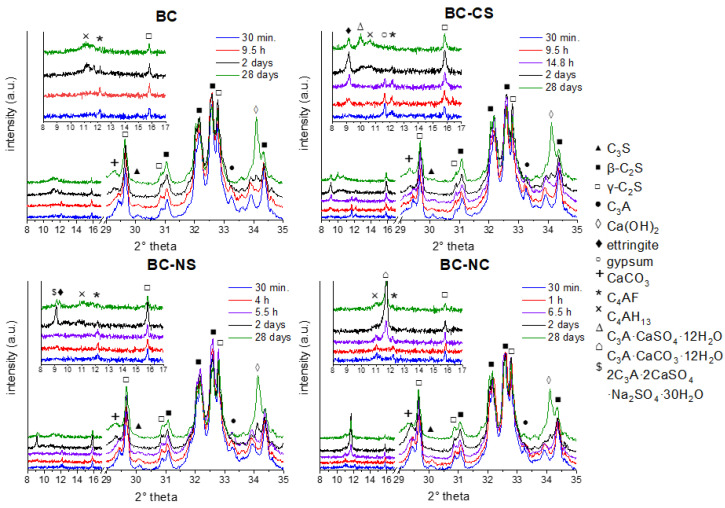
Diffractograms of relevant samples in certain times.

Monitoring of the evolution of the phase composition in individual samples was performed by X-ray diffraction analysis. Figure 2 shows the X-ray diffraction patterns of pure and chemically activated clinker.

#### 3.2.1. BC

During the initial hydration period, at first, the aluminate phases underwent a hydration process and phase conversions. The dissolution of aluminate phases (e.g., C_4_AF and C_3_A) is a part of the initial period of hydration. In the case of pure BC, the main sources of the aluminate phases are C_4_AF and C_3_A. Both aluminate phases converted to the stable form—C_4_AH_13_—which can be observed from the relevant diffractogram, where the intensity of the initial aluminate phase peak decreased concurrently with the increasing C_4_AH_13_ intensity peak. Contrary to the BC with accelerating agents, no sulphate sources were present in the initial mixture; thus, neither ettringite nor its analogues could be formed; hence, the relevant peaks on the diffractogram are not shown. In a more advanced stage of hydration, reactions with silicate phases took place. Initial clinker phases, such as C_3_S, β-C_2_S, underwent dissolution reactions to form the relevant hydration products (C–S–H) and byproducts (CH). The amount of γ-C_2_S, in the case of nonactivated BC, was almost unchanged over time. The increase of portlandite was also observed over time (28 days).

#### 3.2.2. BC-CS

The course of the development of the relevant phases in the case of BC-CS was influenced by the addition of CaSO_4_. Together with the aluminate phases, gypsum participates in the sequence of reactions, where, due to the presence of SO_4_^2−^ groups, the aluminate grains are covered with Aft [32]. The maximum amount of the Aft (alumina, ferric oxide, tri-sulfate) phase was observed after 48 h of hydration. A further decrease is connected to the transformation of AFt into AFm (alumina, ferric oxide, mono-sulfate) and to the formation of the relevant aluminous phase (C_4_AH_13_). Nevertheless, even after 28 days of hydration, there was still a noticeable amount of ettringite present in the sample, which may suggest a higher degree of hydration [33]. The coexistence of AFt, AFm and C_4_AH_13_ was noticed along with total elimination of C_4_AF and C_3_A.

#### 3.2.3. BC-NS

The activation of belite-rich cement by Na_2_SO_4_ introduced a new variable to the creation of alternative transition phases during the transformation of aluminate phases into a hydrated cementitious system. The presence of an ettringite-like structure incorporating sodium sulphate (2C_3_A·2CaSO_4_·Na_2_SO_4_·30H_2_O) was detected next to the AFt in the BC-NS system. The majority of this phase was observed after 48 h of hydration. Its decrease in the advanced stages of hydration was related to the formation of a stable aluminous phase (C_4_AH_13_). A comparison of the degree of ettringite formation between individual systems brings an interesting result. The BC-NS system showed a retarding effect from sodium sulphate concerning the rate of ettringite formation, since it was significantly delayed compared to the BC-CS. A similar effect was stated in the study of Donatello et al. [34].

#### 3.2.4. BC-NC

The occurrence of hydration products, predominantly, the elimination of AFt formation in the case of BC-NC, can be clarified by a deficit of SO_4_^2−^. Moreover, instead of AFm, the relevant carbonate complex (C_3_A·CaCO_3_·12H_2_O) was formed as a predominant transition phase of aluminate grains [10,11]. These grains undergo hydration reactions in subsequent stages of hydration to form a stable aluminous phase, such as C_4_AH_13_. The efficiency of activation via Na_2_CO_3_ can be observed with a reduced amount of γ-C_2_S over time. The activation of γ-C_2_S was investigated by Ashraf [15], who also proved the effectiveness of Na_2_CO_3,_ in reducing gamma-belite over time.

Generally, the possibilities of using activators to help accelerate hydration in the initial phase and activate standard hydraulically inactive components, such as gamma dicalcium silicate, are discussed in [9].

### 3.3. Differential Thermal Analysis

The influence of CS, NS and NC on the hydration process of BC was studied via thermal analysis. The weight loss attributed to the loss of bound water in the C–S–H, Aft, AFm and aluminate hydrated phases was evaluated as a weight loss in the temperature region 0–400 °C. The amount of portlandite was estimated from the weight loss in the temperature range 400–500 °C. The weight loss in the temperature range 500–1000 °C was assigned to the decomposition of carbonates. The total weight loss was used to evaluate the degree of hydration of activated and inactivated BC. Results for the first 48 h of hydration can be seen in Table 3.

#### 3.3.1. BC

**Figure 3:**
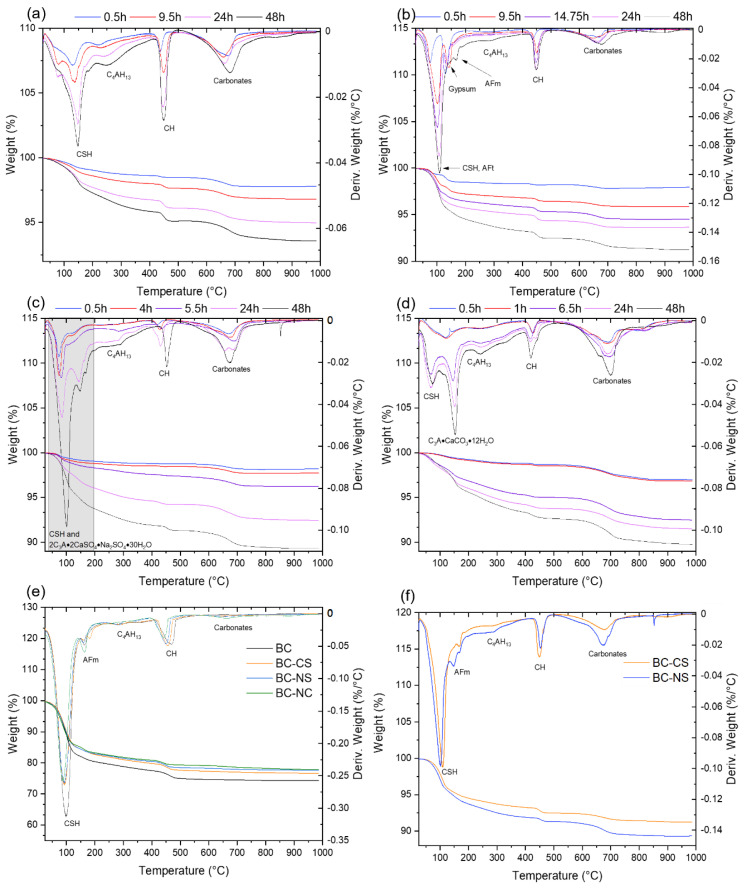
Graphical representation of DTA curves: (**a**) BC, (**b**) BC-CS, (**c**) BC-NS, (**d**) BC-NC, (**e**) 28 days of hydration and (**f**) BC-CS vs. BC-NS.

The dTG curves of the reference BC sample in hydration periods ranging from 30 min to 2 days can be seen in Figure 3a. A broad peak in the reference sample in the temperature range 70–150 °C can be assigned to the loss of weakly bound water from the formed C–S–H phases. The shoulder on the main peak is observable at around 70 °C with the progressing hydration time, and it transforms later on into a separate peak. This low-temperature peak is commonly assigned to the decomposition of amorphous C–S–H gel [35]. This suggests the formation of poor crystalline C–S–H in later stages of hydration, in addition to the initially formed products. As expected, with no SO_4_^2−^ ions present in the cement mixture, the formation of ettringite was not observed. C_4_AH_13_ was formed early in the hydration process, as supported by the XRD results. The C_4_AH_13_ peak was thus clearly observable even after 30 min of hydration at a temperature of 220 °C [36]. With increasing hydration time, the increasing amount of portlandite and carbonated phases was also observable.

#### 3.3.2. BC-CS

The presence of gypsum in the mixture significantly altered the course of hydration. The dTG curves of the BC-CS sample can be seen in Figure 3b. Compared to the reference BC sample, the addition of gypsum caused an increase in weight loss at 0–400 °C in the early phases of hydration. This can be explained by the well-known and extensively described formation of ettringite, which prevents the early formation of hydrated aluminate phases, which cause the flash setting of cement and the retardation of the hydration of clinker minerals [37]. The distinctive peak at 130 °C can be seen up to 24 h after the start of the hydration process, with decreasing intensity as the formation of ettringite progresses. This peak is caused by the dehydration of gypsum. The maximum of the ettringite decomposition peak can be observed at 100 °C with an intensity inversely proportional to the gypsum peak [35]. The formation of the Afm phase becomes apparent after 2 days of hydration as a newly formed peak on the dTG curve with a maximum of around 170 °C. This is supported by the XRD data. The amount of portlandite in the hydrated cement pastes was not significantly influenced by the addition of gypsum. The total weight loss of the samples containing gypsum was higher during the hydration process, which confirms a positive influence on the hydration process and can be directly attributed to a higher amount of hydration products formed.

#### 3.3.3. BC-NS

The dTG results of the Na_2_SO_4_-containing samples can be seen in Figure 3c. Compared to the reference sample, the presence of alkali significantly promoted the formation of CSH in the early hydration stages. The dTG peak in the BC-NS samples measured from 0.5 to 24 h of hydration occurs at 80 °C compared to 130 °C for the reference sample without the presence of an activator. This suggests that alkali ions introduced into the mixture accelerate the hydration of clinker minerals and cause the fast formation of mostly amorphous C–S–H in the early hydration stages [10,11]. The hydration process also substantially differs from the situation where SO_4_^2−^ ions are introduced in the form of gypsum. Instead of ettringite formation in the early hydration stage, large amounts of amorphous C–S–H are formed and a distinct aluminate peak is observed at 280 °C, attributed to the decomposition of C_4_AH_13_. This suggests that the hydration of aluminate phases is not influenced in the same way as with the presence of gypsum and ettringite formation. 

At 48h of hydration time, the dTG diagram becomes almost identical to the BC-CS sample; see Figure 3f. This is in agreement with the XRD diffractogram of the BC-NS samples, which shows a large amount of formed analogue to ettringite with a formula of 2C_3_A·2CaSO_4_·Na_2_SO_4_·30H_2_O. This causes the shift of the low temperature in the dTG peak to 100 °C, similar to the ettringite-containing sample. After 28 days, according to XRD measurement, the amount of 2C_3_A·2CaSO_4_·Na_2_SO_4_·30H_2_O decreases and ettringite appears. However, due to their similar decomposition temperatures and the resulting overlap of their respective dTG peaks, it was not possible to obtain any quantitative information on their respective content in the BC-NS sample.

Na_2_SO_4_ does not act in a similar way to ettringite in altering the aluminate hydration process by forming gradually in the early hydration period. Its formation is shifted to later hydration times and is much more sudden. The amount of formed portlandite decreased significantly compared to both BC and BS-CS samples. The weight loss in the 0–400 °C range related to the decomposition of hydration products and the total weight loss was lower in the first 30 min of hydration, but as the hydration process proceeded, it (BC-NS) eventually exceeded both the BC and BC-CS samples in the first 2 days of hydration, suggesting a positive influence on the early hydration of BC.

#### 3.3.4. BC-NC

The results of a TG/DT analysis of the BC-NC samples are shown in Figure 3d. Due to a lack of SO_4_^2−^ ions, in the first 30 min of hydration, no ettringite was found. BC-NC behaved very similarly to pure the BC sample, as no ettringite was formed. The fact that the amount of hydration products was actually lower in the first 30 min, compared to the BC sample, further proves that the high amount of evolved heat seen during the isothermal calorimetric measurement is indeed only due to Na_2_CO_3_ dissolution hydration and not a rapid formation of hydration products. However, after the initial dissolution. hydration proceeded rather rapidly, forming a significant amount of poorly crystalline C–S–H within 1–24 h of hydration. 

The formation of C_3_A·CaCO_3_·12H_2_O due to the presence of CO_3_^2−^ ions, as confirmed by the XRD analysis of the samples, shows a peak with a maximum around 150 °C in the dTG curves, and this was the main hydration product within the first 48 h of hydration. The amount of portlandite was lower than in the BC and BC-CS samples and higher than in BC-NS. Compared to the BC sample, the weight loss attributed to the decomposition of CSH and Afm phases in BC-NC after only 6.5 h exceeded the amount in the BC sample after 48 h. However, after 48 h, the amount was lower than in BC-NS or even in the BC-CS mixtures. This fact suggests that the addition of Na_2_CO_3_ to BC does not have a significant positive influence on hydration in the first 60 min. The hydration process was greatly accelerated in the 1–24 h hydration time, where large amounts of C_3_A·CaCO_3_·12H_2_O and amorphous C–S–H were formed. However, after the first 48 h, the amount of hydration products was higher in the cement paste activated with Na_2_SO_4_.

This effect can also be seen from the TG/dTG curves of the samples after 28 days of hydration; see Figure 3e, where the total hydration of the BC-NC sample is actually lower than those of all the other samples. After 28 days, the total degree of hydration of BC appears to be lower than that of activated samples, showing that the presence of gypsum, Na_2_SO_4_ or Na_2_CO_3_ has a positive effect mostly only in the very early stages of hydration. These findings are all in good agreement with the data obtained via the isothermal calorimetric investigation of the hydration process.

### 3.4. SEM-EDS Analysis

**Figure 4:**
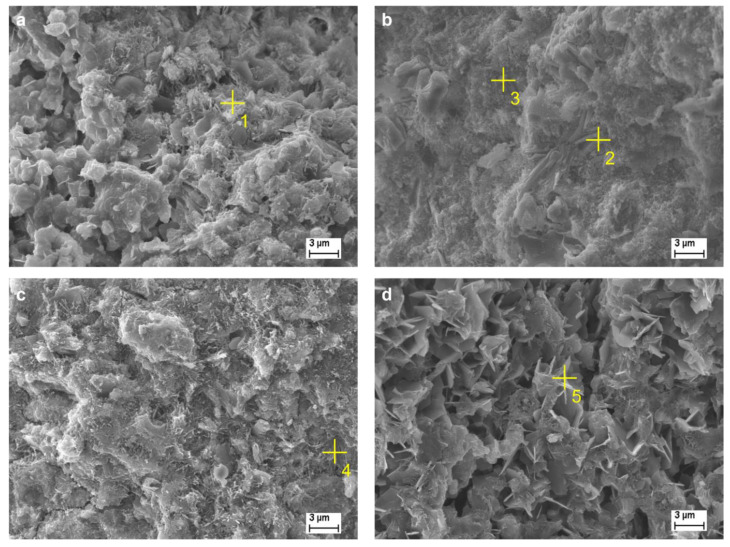
SEM-EDS analysis (28 days); BC (**a**), BC-CS (**b**), BC-NS (**c**), BC-NC (**d**), marked places 1–5 represent the elemental composition.

This section represents evidence of the monitored phases occurring in the fracture surface of the selected samples (Figure 4). The relevant SEM-EDS analysis is illustrated in Table 4.

The pure belite-rich clinker with no additives shows porous structure (Figure 4a), and the evidence of C–S–H phase and portlandite is realized. The presence of portlandite corresponds with the XRD results as well. Point #1 represents the elemental composition of the marked area, where the absence of SO_4_^2−^ results in no sulfur found. The activation of belite-rich cement by CaSO_4_ manifested on the density of the structure and on the phase composition as well. The area marked #2 represents the dense area, supposedly rich in the C–S–H phase. The area marked #3 represents the increased composition of sulfur, which can be affected by the presence of SO_4_^2−^, leading to the formation of the Afm phase, which corresponds to the XRD measurement. The activation by Na_2_SO_4_ influences the structure of belite-cement by the formation of dense structure as well as CaSO_4_. The presence of a combination of SO_4_^2−^ and Na^+^ results in a formation analogous to the Aft phase (area #4), which also corresponds to the XRD measurement (2C_3_A·2CaSO_4_·Na_2_SO_4_·30H_2_O). The formation of the C–S–H phase was observed as well. The structure of belite-rich cement activated by Na_2_CO_3_ showed relatively high porosity. The C–S–H phase and the Afm analogue (C_3_A·CaCO_3_·12H_2_O) were observed (correspondence to the XRD analysis). A higher amount of C can be clarified by the presence of CO_3_^2−^ ions in the alkaline activator.

## 4. Conclusions

The influence of selected alkaline activators in terms of the acceleration of the hydration process in belite-rich cements was assessed. The following conclusions can be drawn:

- The initial stage of hydration in BC is affected by the absence of SO_4_^2−^, which disables ettringite formation; thus, the aluminate phases hydrate very quickly, followed by the formation of stable aluminous phases, such as C_4_AH_13_. The amount of hydraulic nonactive γ-C_2_S remained almost unchanged throughout the hydration period (max. 28 days). The lowest amount of C–S–H phase formed for BC, in contrast to the others, after 48 h.
- The main effect of the activation of belite-rich clinker by CaSO_4_ was the earliest occurrence of ettringite compared to other activators. Moreover, the highest amount of portlandite was detected after 48 h.
- Na_2_SO_4_ showed the most noticeable modification of shape of the silicate peak compared to the other activators. Due to the presence of SO_4_^2^, together with Na^+^, the formation of ettringite and ettringite-like structures was observed. BC-NS showed the greatest formation of C–S–H phase after 48 h, as well as the highest total weight loss.
- The silicate peak accelerated the most in the case of BC-NC due to the presence of CO_3_^2−^, which has the highest dissolution enthalpy. Moreover, the absence of SO_4_^2−^ suppressed the formation of ettringite.
- Based on the measured data, the most appropriate alkaline activator is Na_2_SO_4_, where Na^+^ accelerates the reaction of hydration at the early stage; however, at the same time, SO_4_^2−^ can regulate the undesired fast setting reaction caused by C_3_A, leading to the formation of stable products.

## Figures and Tables

**Table 1:** Chemical composition of a raw mix used for the clinker burning process determined by X-ray fluorescence spectrometry.

Raw Mix	Chemical Composition (wt. %)								
	CaO	SiO_2_	Al_2_O_3_	Fe_2_O_3_	Na_2_O	K_2_O	TiO_2_	MgO	SO_3_
	69.2	19.5	5.7	2.4	0.3	1.6	0.3	0.7	0.3

**Table 2:** Composition of prepared pastes based on belite clinker.

	Constituents (wt. %)				
	Belite Clinker	Water	CaSO_4_·2H_2_O	Na_2_SO_4_	Na_2_CO_3_
BC	66.6	33.3	-	-	-
BC-CS	64.5	32.3	3.2	-	-
BC-NS	64.9	32.4	-	2.7	-
BC-NC	65.3	32.7	-	-	2.0

**Table 3:** Weight loss of selected hydration phases measured by TG-DTA.

		Weight Loss (wt. %)			
		C–S–H	Portlandite	Carbonates	Total wt. Loss
Sample	Hydration Time	0–400 °C	400–500 °C	500–1000 °C	0–1000 °C
BC	0.5	1.325	0.150	0.688	2.21
	9.5	1.961	0.357	0.839	3.20
	24.0	3.190	0.647	1.128	5.02
	48.0	4.089	0.754	1.544	6.43
BC-CS	0.5	1.63	0.106	0.272	2.06
	9.5	3.14	0.378	0.565	4.12
	14.8	4.091	0.528	0.798	5.47
	24.0	4.904	0.688	0.716	6.36
	48.0	6.683	0.782	1.256	8.78
BC-NC	0.5	1.149	0.14	1.695	3.02
	1.0	1.271	0.152	1.702	3.16
	6.5	4.62	0.275	2.518	7.52
	24.0	5.686	0.442	2.264	8.51
	48.0	6.612	0.694	2.859	10.23
BC-NS	0.5	1.166	0.037	0.58	1.81
	4.0	1.424	0.078	0.716	2.26
	5.5	2.358	0.207	1.144	3.77
	24.0	5.309	0.377	1.788	7.56
	48.0	8.028	0.588	1.991	10.68

**Table 4:** SEM-EDS analysis of selected areas.

	Element (at. %)	C	O	Na	Al	Si	S	Ca
Point								
#1		6.57	62.90	-	4.37	6.42	-	19.74
#2		6.62	62.64	-	0.64	10.02	0.26	19.82
#3		5.95	75.64	-	4.86	1.17	2.06	10.32
#4		5.46	58.44	3.00	2.45	7.22	3.14	20.29
#5		8.42	66.29	1.59	4.36	3.91	-	14.79
